# Novel Breast-Specific Long Non-coding RNA LINC00993 Acts as a Tumor Suppressor in Triple-Negative Breast Cancer

**DOI:** 10.3389/fonc.2019.01325

**Published:** 2019-12-17

**Authors:** Shipeng Guo, Lei Jian, Kai Tao, Chen Chen, Haochen Yu, Shengchun Liu

**Affiliations:** Department of Endocrine Breast Surgery, The First Affiliated Hospital of Chongqing Medical University, Chongqing, China

**Keywords:** LINC00993, LncRNA, TNBC, cell cycle, guilt by association

## Abstract

**Background:** Triple-negative breast cancer (TNBC) was characterized by breast cancers that do not express estrogen receptor (ER), progesterone receptor (PR), or human epidermal growth factor receptor (HER)-2 genes. TNBC patients are associated with a shorter median time to relapse and death for the lack of available treatment targets. Long non-coding RNAs (LncRNAs) have been reported to play an important role in the development of TNBC. We identified a novel breast-specific long non-coding RNA LINC00993, but less was known about its expression pattern and functional role in TNBC.

**Methods:** LINC00993 RNA expression was detected across different types of clinical breast cancer samples by using qRT-PCR. Bioinformatic methods “guilt by association” and gene set enrichment analysis (GSEA) were used to predict LINC00993 functions. Subcellular localization of LINC00993 in cells was detected by RNA fluorescence *in situ* hybridization (FISH). Effect of LINC00993 on cell growth was measured by plate colony formation assays, typical growth curve, and an *in vivo* tumor model. Cell cycle analysis was done by flow cytometry analysis. Key cell cycle regulators were detected by Western blot.

**Results:** LINC00993 was largely downregulated in TNBC, and higher expression indicated better outcome. LINC00993 located mainly in the nucleus. LINC00993 suppressed TNBC growth both *in vitro* and *in vivo*. LINC00993 was predicted to be involved in cell cycle pathways by using “guilt by association” and GSEA methods. Key cell cycle regulators like p16^INK4A^, p14^ARF^, p53, and p21 were affected by LINC00993 overexpression.

**Conclusions:** A new breast-specific lincRNA LINC00993 was identified with a tumor-suppressive feature and with prognostic value. This is the first research on LINC00993 function. Our results suggest that controlling LINC00993 level may be beneficial for breast cancer treatment.

## Introduction

Triple-negative breast cancers (TNBCs) are breast cancers that do not express three receptor genes: estrogen receptor (ER), progesterone receptor (PR), and human epidermal growth factor receptor (HER)-2 genes (1). These cancers do not respond to endocrine therapy or other available targeted agents, thus posing a greater clinical treatment challenge (2). Patients diagnosed with TNBC showed a shorter median time to relapse and death (3). However, the mechanism underlying TNBC pathogenesis is not completely understood.

The majority of the human genome are transcribed as non-coding RNAs (ncRNAs) with <2% of the human genome encoding proteins (4). Among these ncRNAs, transcripts of more than 200 nucleotides that are not able to translate into proteins are defined as long non-coding RNAs (lncRNAs). These lncRNAs can be intergenic (long intergenic non-coding RNA, lincRNA), intronic, or natural antisense transcripts. Their transcription are controlled by promoters with divergent enhancers (4). The specific functions of lncRNAs are largely unknown, and they are less conserved across multiple species. LncRNAs can regulate gene expression in *cis*-acting or in *trans*-acting manner (5). LncRNAs are reported to regulate gene expression in diverse biological and pathological contexts, including cancer (6).

By using RNA sequencing (RNA-Seq) and microarray technology, a large number of lncRNAs have been identified to be aberrantly expressed in breast cancers, indicating that those lncRNAs might play a pivotal role in breast cancer (7). Some of these identified lncRNAs were related to breast cancer development (8, 9). Metastasis associated with lung adenocarcinoma transcript 1 (MALAT1) was highly expressed in TNBC tissues, which promoted the proliferation of TNBC cells *in vitro* and promoted TNBC growth and metastasis *in vivo* (10). Antisense oligonucleotides (ASOs) strategy was able to inhibit MALAT1 in a well-characterized mouse model of luminal B breast cancer (11). Those methods were adopted by several researchers to inhibit cancer (12).

Most lncRNAs found in breast cancer are reported to be elevated in tumor tissues compared to normal tissues (13).Functions of lncRNAs are largely unknown, especially for those whose expressions were suppressed in cancers. We previously reported that intergenic lncRNA LINC00993 was greatly downregulated in TNBC (14). A genome-wide transcriptional survey to examine lncRNAs in 995 breast tissue samples found that LINC00993 was the most credible downregulated lncRNA in ER-positive breast cancer compared with ER-negative breast cancer (7). However, the role of LINC00993 in breast cancer is totally unknown.

In this study, we explored the expression pattern of LINC00993 in clinical breast cancer samples and in The Cancer Genome Atlas (TCGA). LINC00993 was found to be breast-specific and was downregulated in breast cancer. We also studied the function of LINC00993 in breast cancer cells both *in vitro* and *in vivo*. Our results suggest that LINC00993 may serve as a tumor suppressor in breast cancer.

## Results

### Guilt by Association Analysis and GSEA Methods Indicated LINC00993's Role in Cell Cycle Regulation

**Figure 1:**
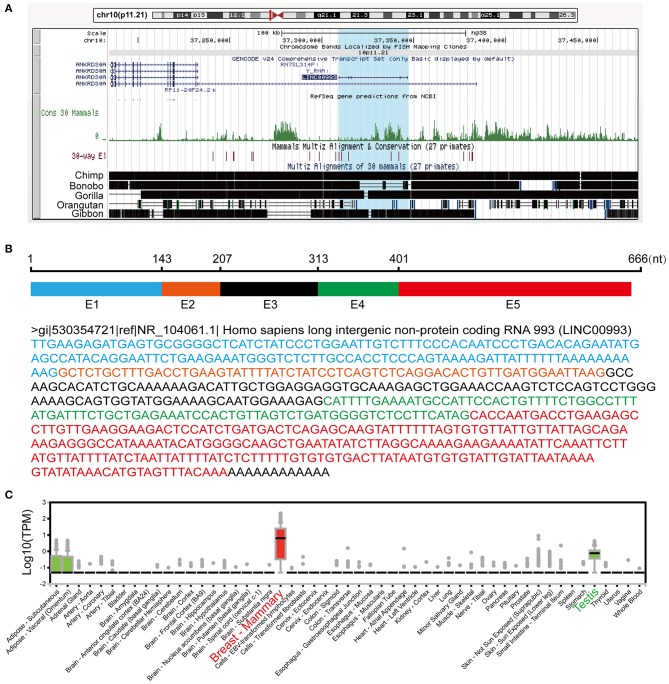
Information about LINC00993 from public websites. **(A)** Location of LINC00993 in human genome. Conservation of LINC00993 in multiple species. **(B)** Construction and sequence of LINC00993. **(C)** Detailed LINC00993 expression in different tissues shown by GTEx data.

LINC00993 is a lincRNA, which maps on chromosome 10p11.21. LINC00993 was not conserved across multiple species, with only chimpanzee sharing the same sequence with human (Figure 1A). The closest gene to LINC00993 is ANKRD30A, which encodes a breast-specific protein (15). LINC00993 is 666 nucleotides (nt) long consisting of five exons (Figure 1B). By analyzing data from the GTEx project (16), LINC00993 was expressed in a tissue-specific pattern, mainly expressed in breast, which is similar to ANKRD30A (Figure 1C). We have previously shown that LINC00993 was downregulated in TNBC tissues, but its function in TNBC is largely unknown.

**Figure 2:**
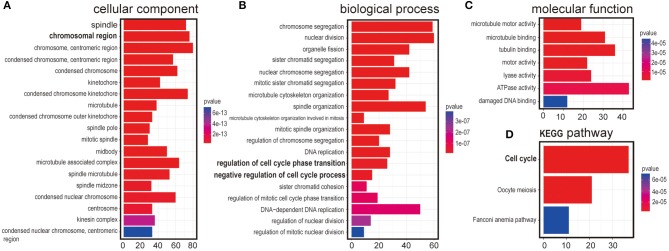
Annotation of LINC00993 via guilt-by-association correlation analysis. Gene Ontology (GO) analysis of LINC00993-related genes in cellular component **(A)**, biological process **(B)**, molecular function **(C)**. **(D)** Kyoto Encyclopedia of Genes and Genomes (KEGG) pathway.

It has been a challenging task to define lncRNA's functions. A “guilt by association” method has been used to predict ncRNA functions (17, 18). To examine the function of LINC00993 in a tissue-specific way, breast cancer data containing 1,208 breast cancer samples from TCGA database (1,096 are from cancer tissues and 112 are from peritumor tissues) were downloaded. We performed correlation analysis between LINC00993 and all 19,547 coding genes in those 1,096 samples by Spearman method. Gene Ontology (GO) analysis and Kyoto Encyclopedia of Genes and Genomes (KEGG) analysis were performed on the top 5% genes (978) according to correlation index. The GO analysis results contain three parts: cellular component, biological process, and molecular function (19). The results showed that LINC00993-related genes took part in the formation of spindle, chromosomal region, and other cellular components, which are mainly located in the nucleus (*p* < 0.001; Figure 2A). Those genes were involved in chromosome segregation, nuclear division, and other biological processes, which also happened in the nucleus (*p* < 0.001; Figure 2B). The molecular function of GO analysis showed that LINC00993-related genes were involved in microtubule motor activity (*p* < 0.001; Figure 2C). The KEGG pathway analysis on those LINC00993-related genes showed that those genes mainly enriched in cell cycle pathway (*p* < 0.001; Figure 2D). As shown in Figure 2B, some of the genes are involved in the regulation of cell cycle-phase transition (*p* < 0.001).

Gene set enrichment analysis (GSEA) is a statistical method to determine if predefined sets of genes are differentially expressed in different phenotypes (20). To analyze which pathway LINC00993 might be involved with, 186 KEGG gene sets from Molecular Signatures Database (MSigDB) were used in GSEA. Most enriched pathways were inhibited by LINC00993 (Figure S1A). Cell cycle was the most significant gene set enriched with a normalized enrichment score as −2.6668 (Supplementary Supplementary Figure S1B). The enrichment score plot showed that LINC00993 negatively regulates cell cycle pathway (Supplementary Figure S1C). For each gene set, a 1,000 times permutation according to gene labels was done to produce a gene set null distribution. As shown in Supplementary Figure S1D, the normal Enrichment Score (ES) for the gene set ranges from −0.4 to 0.4, the ES for gene set cell cycle is −0.674 (*p* < 0.001, FWER < 0.001, FDR < 0.001) (Supplementary Figure S1D). A heat map was drawn based on the expression of genes in the mentioned gene set (Supplementary Figure S1E).

Hallmark gene sets summarize and represent specific well-defined biological states or processes and display coherent expression (21). To examine what potential target genes LINC00993 regulates, 50 hallmark gene sets were analyzed. There were 17 of 50 hallmark gene sets enriched (Supplementary Figure S2A). LINC00993 positively regulates estrogen response (*p* < 0.001, FWER < 0.01, FDR < 0.001), which is consistent with our data that LINC00993 and estrogen receptor (ESR)1 had a strong correlation. The most enriched hallmark data set was E2F targets with a NES at −3.12 (*p* < 0.001, FWER < 0.01, FDR < 0.001) (Supplementary Figure S2B). The running score plot showed that LINC00993 negatively regulated E2F targets (Supplementary Figure S2C). The null distribution plot showed that the result was reliable (Supplementary Figure S2D). A heat map was drawn based on the expression of genes in the E2F targets gene set (Supplementary Figure S2E).

Taken together, those data suggested that LINC00993 might mainly function in the nucleus and was involved in the regulation of the cell cycle.

### LINC00993 Suppressed the Growth of TNBC Cells *in vitro*

**Figure 3:**
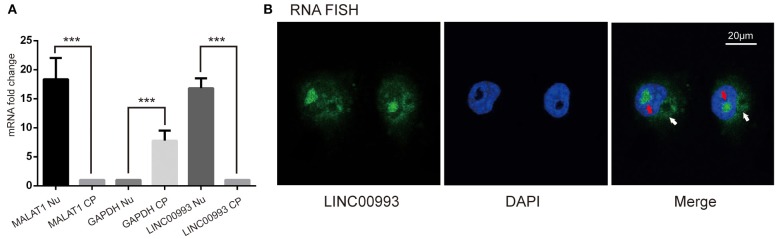
Location of LINC00993 in breast cancer cells. **(A)**. Location of LINC00993 detected by qRT-PCR. Nuclear and cytosolic compartments of MDA-MB-231 cells were extracted. Nu means nuclear, CP means cytoplasm. ***P < 0.001, based on Student's t-test. Data were presented as mean ± SEM. **(B)**. MDA-MB-231 cells were labeled with a LINC00993 probe via RNA fluorescence *in situ* hybridization (FISH) and counterstained with DAPI: 4',6-diamidino-2-phenylindole (DNA to visualize the nucleus). A representative image is shown.

Next, we intended to explore the function of LINC00993 in breast cancer. To choose appropriate cell lines to manipulate the expression of LINC00993, a couple of breast cancer cell lines were detected. LINC00993 was relatively higher in MCF-7 cells when compared to those in MDA-MB-231 cells and BT-549 cells (Supplementary Figure S3A). Lentivirus shRNA method was adopted to knock down LINC00993 in MCF-7, but no significant fold change of LINC00993 was detected in shRNA-stable MCF-7 (Supplementary Figures S3B–F). We thus decided to overexpress LINC00993 in MDA-MB-231 and BT-549, which are two TNBC cell lines that showed a lower LINC00993 expression level. To confirm the location of LINC00993 in TNBC cells, total RNA, cytoplasmic RNA, and nuclear RNA were isolated from MDA-MB-231 cells. Expression of LINC00993 was evaluated in these fractions by qRT-PCR assays. Our results showed that LINC00993 expression was located mainly in the nucleus compared to that in the cytoplasm (16.82 ± 1.69 vs. 1.08 ± 0.03, *p* < 0.01; Figure 3A). Results from RNA fluorescence *in situ* hybridization (FISH) assay also confirmed that most of the LINC00993 located in the nucleus in MDA-MB-231 cells (Figure 3B, red arrow), and some LINC00993 located around nuclear membrane (Figure 3B, white arrow). Those results indicated that LINC00993 mainly resided in the nucleus.

**Figure 4:**
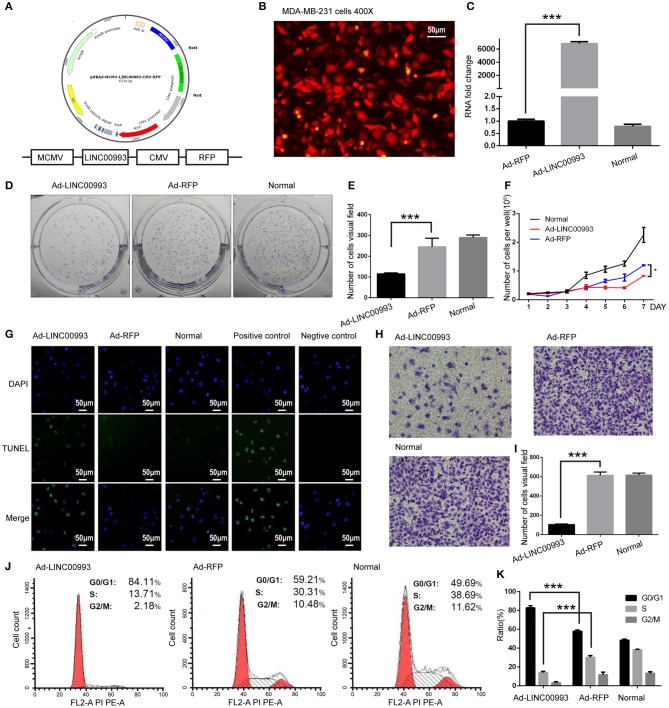
LINC00993 suppresses the growth of triple-negative breast cancer (TNBC) cells *in vitro*. **(A)** Structure of LINC00993 expression plasmid for adenovirus. **(B)** LINC00993 expression adenovirus infection efficiency showed by fluorescence microscope in MDA-MB-231 cells. Original magnification, ×400. Scale bars, 50 μm. **(C)** Expression of LINC00993 detected by qRT-PCR. MDA-MB-231 cells were infected by adenovirus for 24 h, and RNA was extracted. **(D)** Image of clone formation assay. **(E)** Number of clones were counted 2 weeks after plantation. **(F)** MDA-MB-231 cells were planted into 24-well plates. Twenty-four hours later, adenovirus was added to each well. Three wells of cells were digested and counted every 24 h. **(G)** LINC00993 expression caused apoptosis shown by TUNEL assay. Green points reflected apoptosis, and we used DAPI to stain DNA. Positive control cells were treated with DNase I, negative control cells were collected without adding TUNEL reaction buffer. Original magnification, ×100. Scale bars, 50 μm. **(H)** Effect of LINC00993 on cell cycle detected by flow cytometry. **(I)** Flow cytometry cell cycle results shown in a bar plot. **(J)** Invasive ability tested by Transwell assay. Twenty thousand cells were plated in each well, cells were observed after 24 h of incubation. **(K)** Bar plot for the number of cells that migrated across the membrane. ***P < 0.001, based on Student's *t*-test. Data were presented as mean ± SEM.

We then aimed to elucidate the function of LINC00993 in TNBC. Recombinant adenovirus vector expressing LINC00993 under control of a Murine cytomegalovirus (MCMV) promotor and RFP under a Cytomegalovirus (CMV) promotor were constructed (Figure 4A). Fluorescent microscopic analysis of the expression levels of Red fluorescent protein (RFP) confirmed that the infection efficiency was high (Figure 4B), and qRT-PCR assay showed high LINC00993 expression with a 6,000-fold change in RNA expression in adenovirus-infected TNBC cell line MDA-MB-231 (Figure 4C).

To study the role of LINC00993 in cell proliferation, plate colony formation assays were performed with LINC00993 overexpressed MDA-MB-231 cells. Overexpression of LINC00993 decreased the colony formation ability with the number of cell clones dropped from 245 ± 41.58 to 115 ± 5 (*p* < 0.01; Figures 4D,E). LINC00993 decreased the growth rate of MDA-MB-231 (*p* < 0.01; Figure 4F). TUNEL assay showed that LINC00993 overexpression induced MDA-MB-231 cell apoptosis (18 ± 1.0 vs. 3.3 ± 0.5, *p* < 0.01; Figure 4G). Next, flow cytometry analysis of PI-stained MDA-MB-231 cells was used to examine LINC00993's effect on cell cycle. Overexpression of LINC00993 significantly increased the proportion of cells in the G0/G1 phases (84.26 ± 1.86 vs. 56.89 ± 2.06, *p* < 0.001), while decreasing cells in S phases (15.96 ± 8.7 vs. 32.08 ± 1.60, *p* < 0.01; Figures 4H,I), suggesting a potential role of LINC00993 in cell cycle regulation, which was in line with the results from KEGG pathway analysis. As metastasis is an important feature of TNBC, Transwell migration assay was performed to detect the migrating ability of MDA-MB-231 cells. The results showed that LINC00993 strongly suppressed cell migration with less cell transport through membrane (100 ± 8.7 vs. 602 ± 20.56, *p* < 0.001; Figures 4J,K). Similar results were found in BT-549 cells (Supplementary Figures S4A–G). Those results indicated that LINC00993 played a tumor suppressor role in TNBC cells by inhibiting cell proliferation.

### LINC00993 Suppressed the Growth of TNBC Cells *in vivo*

**Figure 5:**
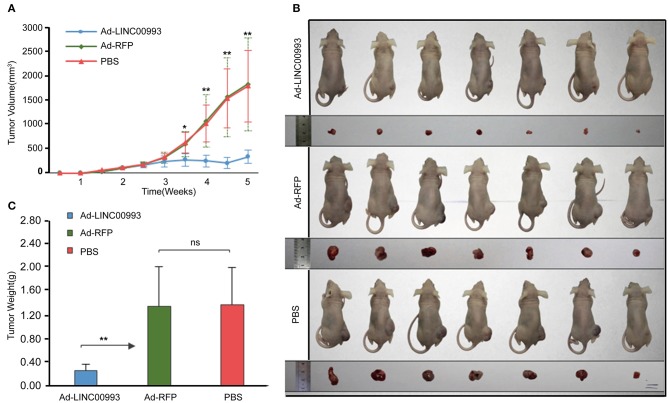
LINC00993 suppresses the growth of triple-negative breast cancer (TNBC) *in vivo*. **(A)** The average tumor volume in Ad-LINC00993 group was much smaller than that in Ad-RFP or PBS group. **(B)** Image of nude mouse burden with breast cancer. Five weeks after cell injection, the mice were sacrificed, and the weight of tumors were examined. **(C)** The average weight of Ad-LINC00993-treated tumors was markedly lower than control. *P < 0.05 and **P < 0.01 based on Student's *t*-test. Data were presented as mean ± SEM.

To confirm the *in vitro* results, nude mice xenograft models of MDA-MB-231 cells were established. About 2 weeks after plantation with tumor size at ~100 mm^3^, the mice were intratumorally injected with Ad-LINC00993, Ad-RFP, or PBS. The volume of the tumor was measured twice a week till 5 weeks. The average tumor volume in Ad-LINC00993 group was much smaller than that in the Ad-RFP group (287.14 ± 68.73 vs. 1,628.57 ± 475.09, *p* < 0.01; Figure 5A). Five weeks after cell injection, the mice were sacrificed and the weight of tumors were examined (Figure 5B). The average weight of Ad-LINC00993-treated tumors was markedly lower than control (0.34 ± 0.05 vs. 1.39 ± 0.27, *p* < 0.01; Figure 5C). The results showed that ectopic expression of LINC00993 in MBA-MD-231 xenografts inhibited tumor growth, thus LINC00993 played a tumor suppressor role in TNBC *in vivo*.

### LINC00993 Functions Through Cell Cycle-Related Genes

Results from KEGG pathway analysis indicated that LINC00993 regulates the cell cycle pathway. Our previous data showed that LINC00993 induced G0/G1 arrest. We were interested to check whether key G0/G1 arrest regulators were changed after LINC00993 overexpression.

**Figure 6:**
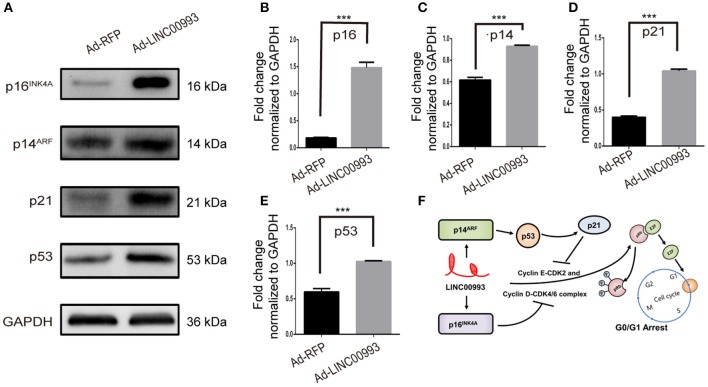
LINC00993 regulates cell cycle-related genes. **(A)** Western blot bands show cell cycle-related proteins infected by LINC00993 overexpression. Integrated optical density (IOD) was measured by Photoshop. The experiment was performed at least three times independently. **(B–E)** Bar graph for Western blot results of p16INK4A, p21, p14ARF, p53 from **(A)**. ***P < 0.001 based on Student's *t*-test. Data were presented as mean ± SEM. **(F)** Schematic model of LINC00993 in breast cancer. LINC00993 upregulates p16INK4A, p14AR, inducing G0/G1 arrest via E2F pathway.

E2F was pivotal in the control of G1 to S phase transition. p16^INK4A^ was reported to inhibit Cyclin D-CDK4/6 complex, which could promote translocation of E2F from cytoplasm to the nucleus. On the other hand, translocation of E2F was also controlled by Cyclin E-CDK2 complex (22, 23). p21 was reported to take part in this process (24). A couple of studies had proved that p14^ARF^-p53-p21 signal pathway was involved in the control of G1/S transition (25, 26). We then decided to detect the expression of those key factors after overexpression of LINC00993. Our results showed that p16^INK4A^ was significantly increased 8-fold (0.18 ± 0.007 vs. 1.48 ± 0.10, *p* < 0.001; Figures 6A,B). p21 expression was raised after LINC00993 expression (0.4 ± 0.02 vs. 1.04 ± 0.02, *p* < 0.001; Figures 6A,D). LINC00993 significantly increased p14^ARF^ to 1.6-fold change (0.62 ± 0.02 vs. 0.93 ± 0.01, *p* < 0.001; Figures 6A,C). At the same time, p53 was also elevated (0.59 ± 0.05 vs. 1.02 ± 0.01, *p* < 0.001; Figures 6A,E). Taken together, our results showed that LINC00993 might inhibit Rb/E2F pathway by upregulating p16^INK4A^, p14^ARF^.

RNA-protein binding assays was performed to investigate potential binding partner interacting with LINC00993. *In vitro*-transcribed biotinylated LINC00993 or its antisense control RNA was incubated with cell extracts from MDA-MB-231, and potential RNA-protein complexes were captured using streptavidin magnetic beads and subjected to mass spectrometry (Supplementary Figure S5A). The most abundant RNA binding protein identified in these pull-downs was heterogeneous nuclear ribonucleoprotein A1 (hnRNPA1) (Supplementary Figure S5B). The binding between hnRNPA1 and LINC00993 was confirmed by Western blotting analysis, showing that sense LINC00993 could pull down hnRNPA1 while its antisense RNA showed less ability to bind hnRNPA1 (Supplementary Figure S5C). Our results clearly demonstrated that LINC00993 interacted with hnRNPA1. hnRNPA1 belongs to the hnRNP family. Some of the hnRNP family members were reported to regulate lncRNAs (27, 28). hnRNPA1 could bind and result in instability of mature p16^INK4A^ and p14^ARF^ mRNAs (29). The lncRNA UCA1 was reported to bind and sequester hnRNPA1, preventing its effect on stability of p16^INK4A^ and p14^ARF^ mRNAs (29). LINC00993 thus might interact with hnRNPA1 this way, regulating the cell cycle-related gene expression. A possible schematic model of LINC00993 function in breast cancer was shown in Figure 6F.

### Validation of LINC00993 Expression in Breast Cancer Samples

**Figure 7:**
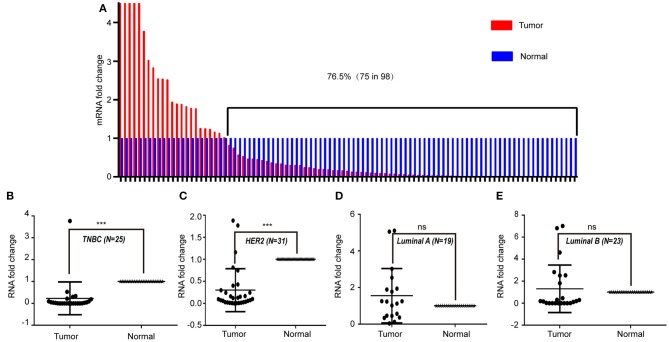
LINC00993 expression in clinical samples. **(A)** LINC00993 expression in 98 tumor and peritumor paired tissues by qRT-PCR. **(B)** LINC00993 expression in triple-negative breast cancer (TNBC) group. **(C)** LINC00993 expression in HER2 group. **(D)** LINC00993 expression in Luminal A group. **(E)** LINC00993 expression in Luminal B group. ns: not significant, ***P < 0.001 based on Student's *t*-test.

To study the expression pattern of LINC00993 in clinical breast cancer samples, LINC00993 was examined in 98 tumor and peritumor paired clinical tissues by qRT-PCR, details of those patients were shown in Table 1. Compared to each paired peritumor tissue, the expression of LINC00993 was suppressed in breast cancer tissues in about 76.5% of the 98 patients (Figure 7A). Subgroup analysis showed that LINC00993 was downregulated in breast cancer tissues when compared to its paired peritumor tissues in TNBC group (*n* = 25, *p* < 0.001; Figure 7B). Similar results were observed in HER2 group (*n* = 31, *p* < 0.001; Figure 7C). No significant changes of LINC00993 levels were found in Luminal A group (*n* = 19, *p* > 0.05) and Luminal B group (*n* = 23, *p* > 0.05; Figures 7D,E).

**Table 1:** Clinicopathological features of the study cohort (*n* = 98).

**Parameter**	**No. (%)**	**LINC00993 expression level**		***p*-value**
		**High in cancer (n = 23) no. (%)**	**Low in cancer (*n* = 75) no. (%)**	
**Age (year)**				
≤ 40	14 (14.3)	5 (21.7)	9 (12.0)	
>40	84 (85.7)	18 (78.3)	66 (88.0)	0.243
**Menopause**				
Yes	48 (49.0)	10 (43.5)	38 (50.7)	
No	50 (51.0)	13 (56.5)	37 (49.3)	0.546
**Subtypes of Cancer**				
Ductal	94 (96.0)	22 (95.7)	72 (96.0)	
Lobular	3 (3.0)	1 (4.3)	2 (2.7)	
Others	1 (1.0)	0 (0.0)	1 (1.3)	0.791
**Tumor Size**				
≤ 2 cm	33 (33.7)	9 (39.1)	24 (32.0)	
>2 cm ≤ 4 cm	59 (60.2)	14 (60.9)	45 (60.0)	
>4 cm	6 (6.1)	0 (0.0)	6 (8.0)	0.349
**Clinical nodal status**				
Positive	46 (46.9)	14 (60.9)	32 (42.7)	
Negative	52 (53.1)	9 (39.1)	43 (57.3)	0.126
**Histological Grade**				
I	7 (7.1)	2 (8.7)	5 (6.7)	
II	73 (74.5)	20 (87.0)	53 (70.7)	
III	18 (18.4)	1 (4.3)	17 (22.6)	0.139
**Pathology Molecular Typing**				
Luminal A	19 (19.4)	12 (52.2)	7 (9.3)	
Luminal B	23 (23.5)	7 (30.4)	16 (21.3)	
HER2	31 (31.6)	3 (13.1)	28 (37.3)	
Triple-neg	25 (25.5)	1 (4.3)	24 (32.1)	<0.001
**Neoadjuvant Chemotherapy**				
Yes	18 (18.4)	5 (21.7)	13 (17.3)	
No	80 (81.6)	18 (78.3)	62 (82.7)	0.633
**ER status**				
Positive	41 (41.8)	19 (82.6)	22 (29.3)	
Negative	57 (58.2)	4 (17.4)	53 (70.7)	<0.001
**PR status**				
Positive	40 (40.8)	18 (78.3)	22 (29.3)	
Negative	58 (59.2)	5 (21.7)	53 (70.7)	<0.001
**HER2 status**				
Positive	43 (43.9)	8 (34.8)	35 (46.7)	
Negative	55 (56.1)	15 (65.2)	40 (53.3)	0.315
**Ki67 status**				
<14%	29 (40.8)	14 (60.9)	15 (20.0)	
≥14%	69 (59.2)	9 (39.1)	60 (80.0)	<0.001

*ER, estrogen receptor; PR, progesterone receptor; HER2, human epidermal growth factor receptor 2. Fisher's exact test*.

**Figure 8:**
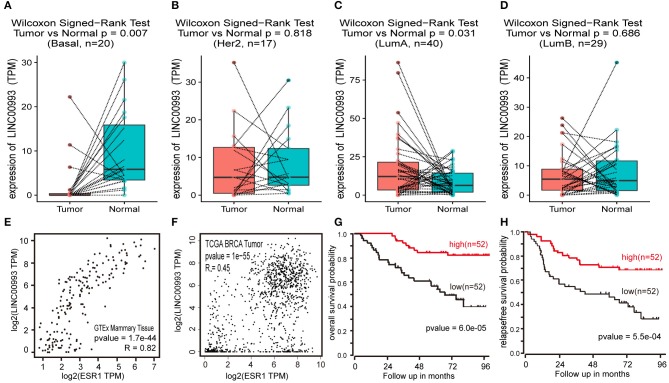
LINC00993 expression in The Cancer Genome Atlas (TCGA) database. **(A)** Paired boxplot of LINC00993 expression in triple-negative breast cancer (TNBC) group. **(B)** Paired boxplot of LINC00993 expression in HER2 group. **(C)** Paired boxplot of LINC00993 expression in Luminal A group. **(D)** Paired boxplot of LINC00993 expression in Luminal B group. **(E)** Correlation analysis between LINC00993 and estrogen receptor (ESR)1 expression in GTEx mammary tissues using the Spearman method. **(F)** Correlation analysis between LINC00993 and ESR1 expression in TCGA BRCA tumor tissues using the Spearman method. **(G)** Overall survival Kaplan-Meier analysis was performed using the R2 database. **(H)** Relapse-free survival Kaplan-Meier analysis was performed using the R2 database.

To confirm these findings, Breast Invasive Carcinoma (BRCA) data set from TCGA project was analyzed. Breast cancer subtype data were generated by using PAM50 predictor bioclassifier (30). Paired tumor and peritumor samples were extracted, and LINC00993 was found markedly downregulated in basal-like group compared to that in Normal group (*n* = 20, *p* = 0.007; Figure 8A). No significant difference was found in Her2 group (*n* = 17, *p* = 0.818; Figure 8B) and in Luminal B group (*n* = 29, *p* < 0.0001; Figure 8D). However, in Luminal A group (*n* = 330), LINC00993 was slightly elevated (*n* = 40, *p* = 0.031; Figure 8C).

As LINC00993 was downregulated in TNBC group but upregulated slightly in Luminal A group, we tested whether LINC00993 expression correlates with ER status or not. ER was encoded by ESR1 gene, a correlation analysis was carried out by using GEPIA. LINC00993 and ESR1 showed a high positive correlation index at 0.82 in GTEx mammary tissues (*p* < 0.0001; Figure 8E). In TCGA breast cancer tissues, they still showed a high positive correlation index at 0.45 (*p* < 0.0001; Figure 8F).

Due to the low expression level of LINC00993 in breast cancer, a Kaplan-Meier survival analysis was performed based on LINC00993 gene expression by using R2 database (http://r2.amc.nl). The overall survival curves suggested that breast cancer patients benefited from high LINC00993 expression (*p* < 0.0001; Figure 8G). The relapse-free survival analysis showed a similar result that a higher LINC00993 expression had a better outcome (*p* < 0.001; Figure 8H). Our results indicated that LINC00993 was strongly downregulated in TNBC, and its expression in breast cancer might serve as a prognostic biomarker. Taken all these results together, LINC00993 was breast-specific and was identified with a tumor suppressive feature and with prognostic value, suggesting that controlling LINC00993 level may be helpful for breast cancer treatment.

## Discussion

Here we identified a novel breast-specific long non-coding RNA LINC00993 with tumor suppressive feature. We proved that LINC00993 was largely downregulated in breast cancer, especially in TNBC. LINC00993 suppressed breast cancer growth by causing G0/G1 arrest. We further showed that LINC00993 regulated key cell cycle-related genes such as p16^INK4A^, p14^ARF^, p21, and p53. As E2F played a significant role in regulation of G1 to S phase transition, LINC00993 could regulate translocation of E2F through two possible pathways, one is p16^INK4A^-CDK4/6- Rb/E2, the other is p14^ARF^- p53- p21-CDK2- Rb/E2 (Figure 6F). One interesting thing is that p16^INK4A^ is a tumor suppressor protein encoded by the CDKN2A gene, while p14^ARF^ is an alternate reading frame protein product of the CDKN2A locus. Because they were upregulated by LINC00993, future studies are needed to find out how LINC00993 affects p16^INK4A^, p14^ARF^ expression. One possible mechanism is that LINC00993 might regulate CDKN2A gene transcription. Based on our findings, LINC00993 showed the potential to be a novel cancer therapeutic target.

Although LINC00993 was greatly downregulated in TNBC, how it was regulated remains unknown. By analyzing 862 pieces of breast-related ChIP-seq data sets from Cistrome database, FOXA1 binding peaks were found around LINC00993 gene in several data sets, indicating that FOXA1 might regulate LINC00993 expression (data not shown). FOXA1 could maintain luminal related genes and suppress basal-like genes (31). Expression of FOXA1 in luminal cancer was much higher than that in basal-like breast cancer. FOXA1 is the primary determinant of ER binding and transcriptional activity in breast cancer cells (32). This might explain why LINC00993 correlated with ER but did not affect each other (data not shown). They might be downstream elements of FOXA1. FOXA1 might serve as a key regulator of LINC00993, but how FOXA1 regulates LINC00993 remains to be investigated.

A large proportion of the human and mouse genome is transcribed as ncRNAs. Although some lncRNAs are verified to be functional, most of them remain largely unknown. Researchers have adopted guilt-by-association method to annotate lncRNAs (8). Although there are some database annotate ncRNAs this way, none of them offered information for LINC00993 (33). In this article, we combined two different bioinformatical method to annotate breast-specific LINC00993. First, we got a gene list containing genes mostly correlated with LINC00993 in expression. We did GO and KEGG analysis based on the gene list. Our results showed a strong relationship between LINC00993 and cell cycle process. We then used another method to annotate LINC00993, which we called single gene GSEA. Usually, GESA analysis needs at least two phenotypes to rank gene list. Here, we created the needed two groups by separating 1,096 breast cancer samples according to LINC00993 expression. To make the result more accurate, we set a threshold at 10%, the top 10% as LINC00993 high group and bottom 10% as low group. Our results showed that LINC00993 was involved in cell cycle pathway, and it might regulate G1/S transition (34). Those results were confirmed by wet lab experiments. Meanwhile, our GSEA analysis offered some other information about LINC00993, like chromosomal segregation, cilium pathway, and sperm formation. Considering that most of the lncRNAs are tissue-specific and expressed with a low abundance, annotation of them is a hard task. Based on RNA-Seq data from TCGA, “guilt by association” and GSEA method might be potential tools in exploring functions of cancer-related lncRNAs. However, more experiments were needed to evaluate the feasibility.

In conclusion, LINC00993 is a breast-specific lincRNA, which was downregulated in breast cancer, especially in TNBC. LINC00993 suppresses TNBC growth both *in vitro* and *in vivo*. Our findings here help with the understanding of TNBC development. LINC00993 showed the potential to be a novel cancer therapeutic target.

## Materials and Methods

### Cell Culture

The human breast cancer cell line MDA-MB-231 and BT-549 were purchased from the cell bank of Shanghai Institute of Biological Sciences, Chinese Academy of Science. These cells were maintained with DMEM basal medium containing 10% fetal bovine serum in a humidified incubator with 5% CO_2_ at 37°C.

### RNA Isolation and Quantitative Real-Time PCR (qRT-PCR)

Total RNA was isolated using TRIzol reagent (Ambion, 15596-026, USA) according to manufacturer instructions. PrimeScript™ RT reagent Kit (Takara, RR037A) was used to synthesize the first-strand cDNA from an equal amount of the RNA sample. qRT-PCR was done by using SYBR® Premix Ex Taq® II (Takara, RR820A). The relative expression of mRNA was calculated with the 2^−ΔΔ*T*^ method. Each sample was repeated in triplicate. qRT-PCR data were analyzed and converted to relative fold changes. The following qRT-PCR assay primer sequences were used: LINC00993: 5′-AGTGCGGGGCTCATCTATCC (forward) and 5′TTACTGGGAGGTGGCAAGAG (reverse); Glyceraldehyde 3-phosphate dehydrogenase (GAPDH), 5′-CTCTGCTCCTCCTGTTCGAC (forward) and 5′ GCGCCCAATACGACCAAATC (reverse).

### Plasmid Construction and Adenovirus Production and Infection

The shuttle plasmid of adenovirus was constructed by inserting synthesized LINC00993 sequence into pHBAd-MCMV-RFP vector (Hanbio, Shanghai). The construction was done by using Gibson Assembly method with the restriction enzyme sites *Not* I and *Nsi* I. The shuttle plasmid was confirmed by sequencing. The shuttle plasmids were transfected into HEK293 cells together with the adenovirus packaging plasmid pBHGloxdelE13cre using the Lipofectamine® 2000 DNA Transfection Reagent (Thermo Fisher Scientific). Six days later, first-generation adenoviruses were harvested. All adenoviruses were amplified in HEK293 cells and purified by ultracentrifugation on cesium chloride (CsCl) gradients. The titer of adenovirus was determined by TCID50 method. For MDA-MB-231 cell infection, the multiplicity of infection (MOI) equals to 40 was adopted after multiple tests.

### Flow Cytometry Analysis

Flow cytometry analysis was used to analyze cell cycle. In brief, after transfection with adenovirus vectors for 48 h, cells were trypsinized and washed twice with ice-cold PBS, and then fixed with 75% ethanol at 4°C overnight. The fixed cells were suspended in propridium iodide (PI, 100 mg/ml) or RNase (10 mg/ml) or PBS, and incubated at 37°C for 30 min in the dark. All samples were assessed with FACScan system (BD Biosciences, USA). Data were analyzed by Cell Quest software (BD Biosciences, USA). Measurements were repeated independently three times.

### Transwell Assay

Cell invasion capability was measured using Corning BioCoat™ 24-well-cell permeable supports (8 μm pore size, Corning, USA). Briefly, the upper chamber of the Transwell was coated with 150 μl diluted BD Matrigel™ Basement membrane Matrix (BD, USA) gel solution (200 μg/ml) at 37°C for 3 h. Medium (800 μl) with 10% FBS was added to the lower chamber, and 2 × 10^4^ cells were then plated in the upper chamber with 100 μl serum-free medium. After incubation at 37°C with 5% CO_2_ for 24 h, cells were fixed with methanol and acetic acid (3:1) and stained with Giemsa staining (SolarBio, Beijing, China). Cells remaining on the upper membrane were gently removed by wiping with cotton swabs. Cell invasion was assessed by counting stained cells under a microscope on five random fields. Three replicates were performed.

### TUNEL Assays

To analyze cell apoptosis, terminal deoxynucleotidyl transferase-mediated dUTP nick-end labeling (TUNEL) assays were performed with One Step TUNEL Apoptosis Assay Kit (FITC, Beyotime, China) according to the manufacturer's instructions. DNase I-treated cells were used as positive control. FITC-labeled apoptotic cells were observed under a fluorescence microscope.

### CCK-8 Assay, Cell Growth Curve, and Colony Formation Assays

Cell viability was measured using Cell Counting Kit-8 (CCK-8) (Dojindo, Japan). For cell growth assay, cells were plated in a 24-cell plate, and after adenovirus infection, three wells of cells were randomly picked up, digested, and counted. For the colony formation assay, 1–3 × 10^3^ cells were plated in each well of a six-well plate and incubated at 37°C for 1–2 weeks. The cells were fixed with 4% paraformaldehyde and stained with 1% crystal violet (Sigma-Aldrich). Cell colonies were counted and analyzed.

### *In vivo* Tumor Model

The animal experimental protocols were approved by the Committee on Ethical Use of Animal of the First Affiliated Hospital of Chongqing Medical University. Female BALB/c nude mice of 4~6 weeks old were maintained under SPF (specific pathogen-free) conditions in the Experimental Animal Department of the Chongqing Medical University. The mice (*n* = 21) were randomized and grouped with seven each. MDA-MB-231 cells (2 × 10^6^) in 200 μl serum-free medium were subcutaneously injected to the right dorsal flank of the mice. When ~100 mm^3^ tumors were observed, the mice were received intratumoral injection with 10^9^ plaque-forming units (PFU) Ad-LINC00993, Ad-RFP, or PBS in a total volume of 50 μl. Delivery of viral vectors or PBS started at the second week and was repeated every 2 weeks. Five weeks after cell injection, the mice were sacrificed. The total volumes of tumors were assessed twice a week using a caliper and was calculated with the longest diameter (X) as well as the shortest diameter (Y) using the formula: X ^*^ Y^2^/2. The weight of tumor was measured after mice were sacrificed.

### RNA Pulldown and Mass Spectrometry

*In vitro*, biotin-labeled RNAs were transcribed with the Biotin RNA Labeling Mix (Roche) and T7 RNA polymerase, treated with 2 μl RNase-free DNase I at 37°C for 15 min to remove DNA and add 2 μl 0.2 M EDTA (pH = 8.0) to stop the reaction. Biotinylated RNA (1 μg) in RNA structure buffer (10 mM Tris pH 7, 0.1 M KCl, 10 mM MgCl_2_) was heated to 95°C for 2 min, on ice for 3 min, and then left at room temperature (RT) for 30 min to allow proper secondary structure formation. Folded RNA was then mixed with human MDA-MB-231 cell extract (containing 1 mg proteins) in 500 μl RIP buffer and then incubated at RT for 1 h. Washed streptavidin agarose beads (50 μl) (Invitrogen, Cat. No. SA10004) was added to each binding reaction and further incubated at RT for another 1 h. Beads were washed briefly with RIP buffer three times and boiled in SDS buffer. Then, the retrieved proteins were detected by Western blot or resolved in gradient gel electrophoresis followed by mass spectrometry (MS) identification.

### Western Blot

Cell lysates were separated by 10% sodium dodecyl sulfate-polyacrylamide gel electrophoresis (SDS-PAGE). Autoradiograms were quantified by densitometry (Quantity One software; Bio-Rad). GAPDH antibody was used as control. p16^INK4A^, p14^ARF^, p21, p53, and GAPDH antibody were purchased from Proteintech (Wuhan, China).

### Bioinformatics Methods and Data Analysis

Correlation analysis was done by using GEPIA using Spearman method (35). Survival analysis was carried out by using R2 (http://r2.amc.nl), a Genomics Analysis and Visualization Platform. BRCA data were downloaded from TCGA by using GDC Data Transfer Tool (36). Those data were arranged by using R language and were and normalized by “DESeq2” package (37). GSEA analysis requires at least two different phenotypes. One thousand ninety-six BRCA RNA-Seq data were downloaded from TCGA by using GDC tools. Those samples were arranged according to LINC00993 expression. The top 10% (110) of the data that expressed a high level of LINC00993 were chosen as high group; the bottom 10% (110) as low group. GSEA analysis was performed by using the two groups. GSEA analysis was done by using the R script from Board Company. The statistical significance of comparisons between two groups was determined with Student's *t*-test. *P* < 0.05 were considered statistically significant.

### Patients and Tumor Specimens

The study was approved by the Ethics Committee of Chongqing Medical University, and it was performed in compliance with the Declaration of Helsinki principles. The written informed consent was obtained from all patients. Breast cancer samples from 98 patients consecutively from July 2015 until Jun 2018 were analyzed in this study. Those patients underwent surgical resection of the breast cancer at the First Affiliated Hospital of Chongqing Medical University. Tumor specimens were from surgical resection and frozen in liquid nitrogen for 1 h within 30 min and then stored in −80°C for long time preservation.

### Data Analysis

The values represent the mean ± SEM. The statistical significance of comparisons between two groups was determined with Student's *t*-test. *P* < 0.05 were considered statistically significant.

## Data Availability Statement

Publicly available datasets were analyzed in this study. This data can be found here: https://portal.gdc.cancer.gov/; https://gtexportal.org/; http://gepia.cancer-pku.cn/index.html; https://hgserver1.amc.nl/cgi-bin/r2/main.cgi.

## Ethics Statement

The studies involving human participants were reviewed and approved by Human Research Ethics Committee of the First Affiliated Hospital of Chongqing Medical University. The patients/participants provided their written informed consent to participate in this study. This animal study was reviewed and approved by Animal Experimentation Ethics Committee of Chongqing Medical University.

## Author Contributions

SG, LJ, KT, and CC performed the research. SL designed the research study. SG contributed essential reagents or tools. SG, LJ, HY, and CC analyzed the data. SG and SL wrote the paper. All authors have read and approved the final manuscript.

### Conflict of Interest

The authors declare that the research was conducted in the absence of any commercial or financial relationships that could be construed as a potential conflict of interest.
