# Supplementary material for: Novel Breast-Specific Long Non-coding RNA LINC00993 Acts as a Tumor Suppressor in Triple-Negative Breast Cancer
Source: Front Oncol. 2019 Dec 17;9:1325. doi: 10.3389/fonc.2019.01325 (PMC6928780; doi:10.3389/fonc.2019.01325)
Supplement: Supplementary file 1 [file Data_Sheet_1.pdf]

Supplementary Material

A

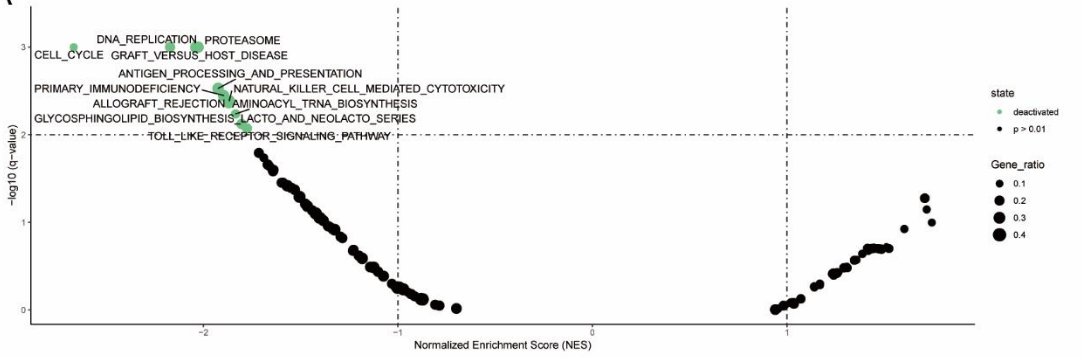

B

GS	SIZE	ES	NES	NOM p-val	FDR_q_val	FWER p-val	Gene_ratio
KEGG_CELL_CYCLE	124	-0.67412	-2.6668	0	0	0	0.115
KEGG_DNA_REPLICATION	36	-0.70096	-2.1715	0	0	0	0.214
KEGG_GRAFT_VERSUS_HOST_DISEASE	37	-0.65331	-2.038	0	0	0	0.291
KEGG_PROTEASOME	43	-0.63103	-2.0275	0	0	0	0.286
KEGG_ANTIGEN_PROCESSING_AND_PRESENTATION	79	-0.53524	-1.9235	0	0.0019537	0.007	0.291
KEGG_PRIMARY_IMMUNODEFICIENCY	35	-0.61232	-1.8956	0	0.0025629	0.011	0.281
KEGG_ALLOGRAFT_REJECTION	35	-0.60665	-1.8698	0.00231	0.0033137	0.017	0.267
KEGG_NATURAL_KILLER_CELL_MEDIATED_CYTOTOXICITY	130	-0.46773	-1.8671	0	0.0030949	0.018	0.285
KEGG_AMINOACYL_TRNA_BIOSYNTHESIS	41	-0.56722	-1.8341	0	0.0047849	0.03	0.161
KEGG_GLYCOSPHINGOLIPID_BIOSYNTHESIS_LACTO_AND_NEOLACTO_SERIES	25	-0.62316	-1.8037	0.002358	0.006542	0.044	0.221
KEGG_TOLL_LIKE_RECEPTOR_SIGNALING_PATHWAY	101	-0.4663	-1.7761	0	0.0074561	0.055	0.205

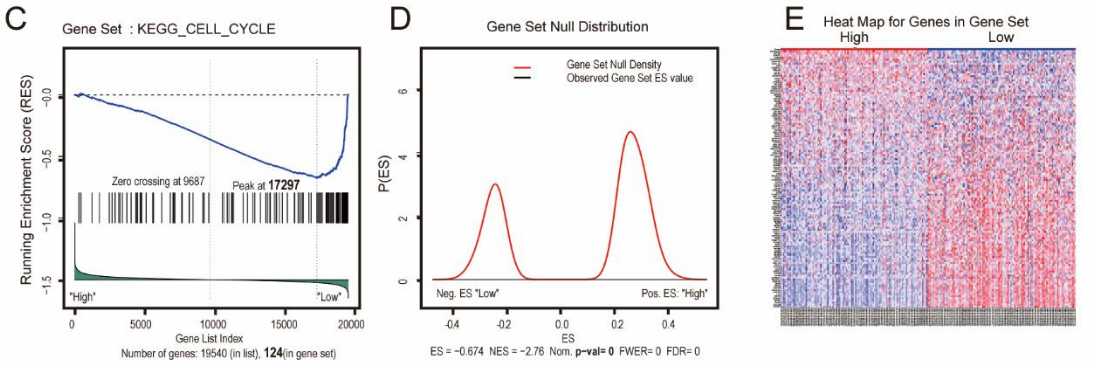

Supplementary Figure S1. Annotation of LINC00993 by GSEA. (A). Volcano plot for enriched KEGG gene sets for LINC00993 GSEA (FDR_q_val < 0.01). (B). Detailed information table for enriched gene sets of KEGG analysis. GS: Gene Sets. ES: Enrichment score. NES: Normalized enrichment score. NOM p-val: Nominal p-value (from the null distribution of the gene set). FDR q-val: False discovery rate q-values. FWER p-val: Family wise error rate p-values. (C) Enrichment score plot for gene set KEGG_CELL_CYCLE. (D) Gene set null distribution was drew by doing a 1000 times permutation. Permutations were done by reshuffling gene labels each time. (E)Heat map for genes in gene set KEGG_CELL_CYCLE.

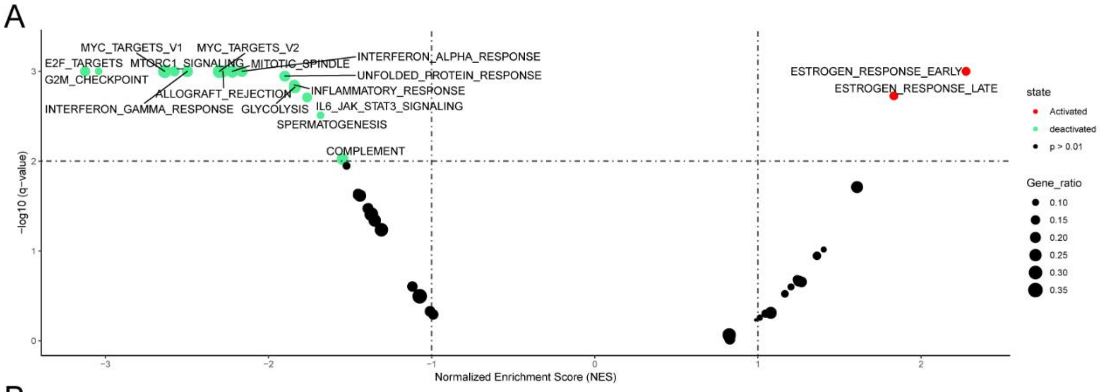

B

GS	SIZE	ES	NES	NOM p-val	FDR_q_val	FWER p-val	Gene_ratio
HALLMARK_ESTROGEN_RESPONSE_EARLY	196	0.58753	2.2757	0	0	0	0.134
HALLMARK_ESTROGEN_RESPONSE_LATE	197	0.47488	1.8334	0	0.00087646	0.003	0.134
HALLMARK_E2F_TARGETS	196	-0.75712	-3.1232	0	0	0	0.174
HALLMARK_G2M_CHECKPOINT	194	-0.73017	-3.0415	0	0	0	0.109
HALLMARK_MYC_TARGETS_V1	197	-0.63843	-2.6358	0	0	0	0.288
HALLMARK_MTORC1_SIGNALING	197	-0.62	-2.5759	0	0	0	0.178
HALLMARK_INTERFERON_GAMMA_RESPONSE	198	-0.6015	-2.497	0	0	0	0.209
HALLMARK_MYC_TARGETS_V2	58	-0.67103	-2.3007	0	0	0	0.289
HALLMARK_ALLOGRAFT_REJECTION	199	-0.54557	-2.2795	0	0	0	0.235
HALLMARK_MITOTIC_SPINDLE	198	-0.53251	-2.2215	0	0	0	0.251
HALLMARK_INTERFERON_ALPHA_RESPONSE	94	-0.57166	-2.1623	0	0	0	0.199
HALLMARK_UNFOLDED_PROTEIN_RESPONSE	109	-0.48923	-1.8992	0	0.000128	0.001	0.194
HALLMARK_INFLAMMATORY_RESPONSE	196	-0.43853	-1.8414	0	0.00041651	0.003	0.186
HALLMARK_GLYCOLYSIS	197	-0.44418	-1.8386	0	0.00050301	0.004	0.23
HALLMARK_IL6_JAK_STAT3_SIGNALING	87	-0.47033	-1.7627	0	0.00094792	0.008	0.167
HALLMARK_SPERMATOGENESIS	133	-0.42297	-1.6811	0	0.0020749	0.017	0.11
HALLMARK_COMPLEMENT	195	-0.37232	-1.5453	0	0.0084425	0.069	0.222

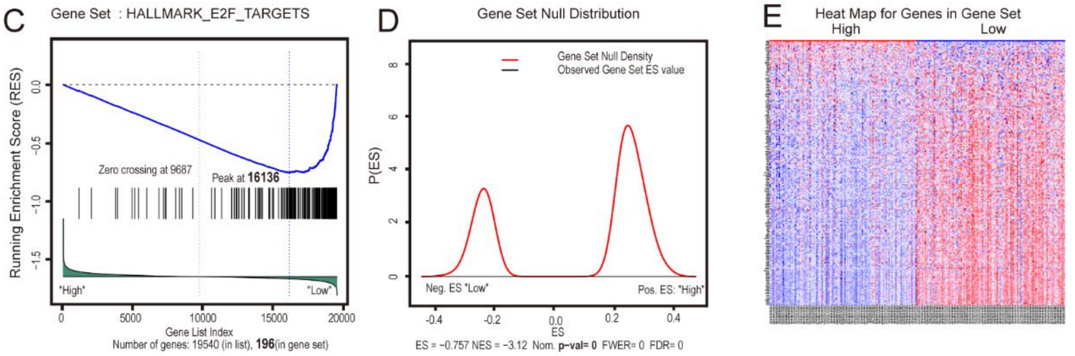

Supplementary Figure S2. GSEA results of Hallmarks gene sets analysis of LINC00993 in breast cancer. (A) Volcano plot for enriched Hallmarks gene sets for LINC00993 GSEA (FDR_q_val < 0.01). (B) Detailed information table for enriched gene sets of Hallmarks analysis. (C) Enrichment score plot for gene set HALLMARK_E2F_TARGETS. (D) Gene set null distribution was drew by doing a 1000 times permutation. Permutations were done by reshuffling gene labels each time. (E)Heat map for genes in gene set HALLMARK_E2F_TARGETS.

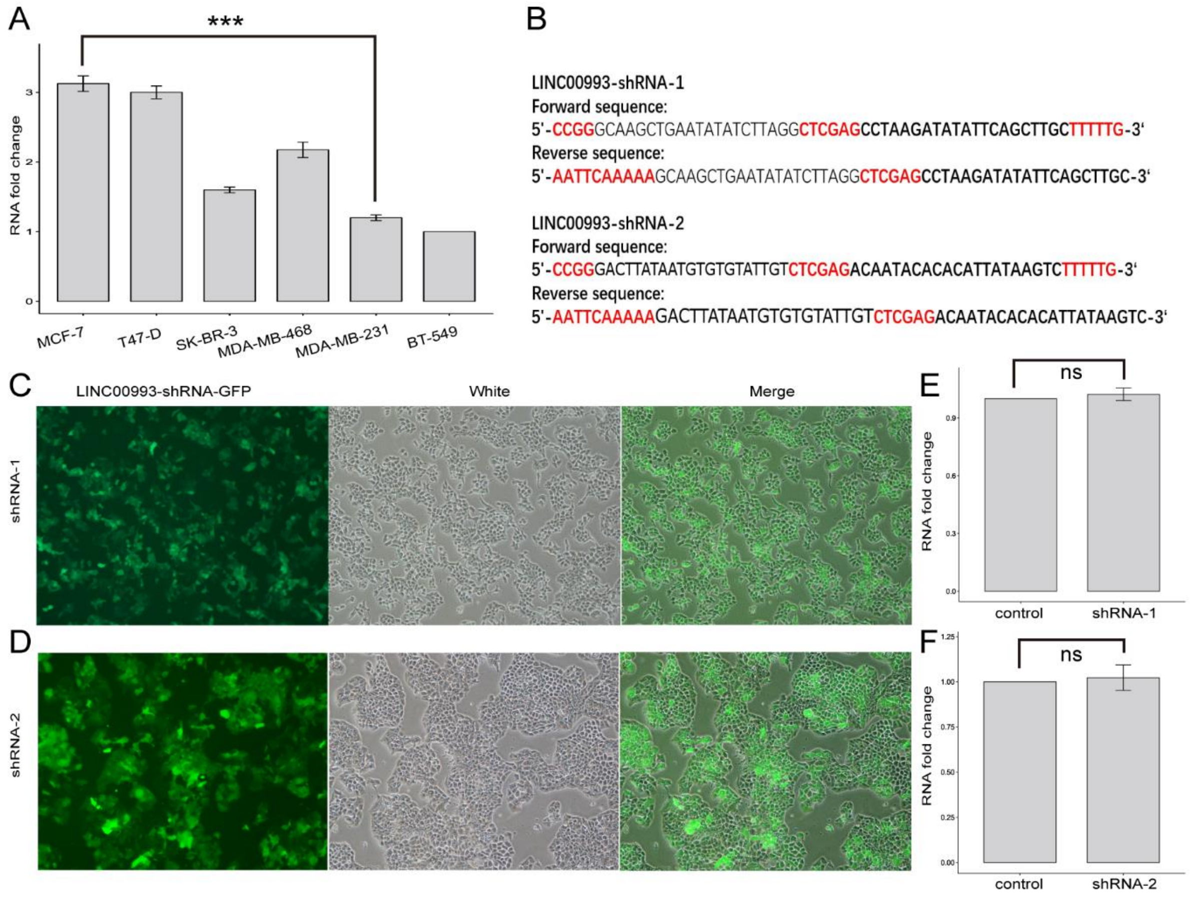

Supplementary Figure S3. Cell line for the experiment. (A) Bar plot showing the expression of LINC000993 in different breast cell lines detected by qRT-PCR. (B) Sequence information of two different shRNAs. Forward and Reverse primers were listed (C-D) shRNA lentivirus infection efficiency showed by Fluorescence microscope in MCF-7 cells. (E-F) Expression of LINC00993 detected by qRT-PCR in different shRNA infected MCF-7 cell lines. ns: not significant, ***P < 0.001 based on Student's t-test.

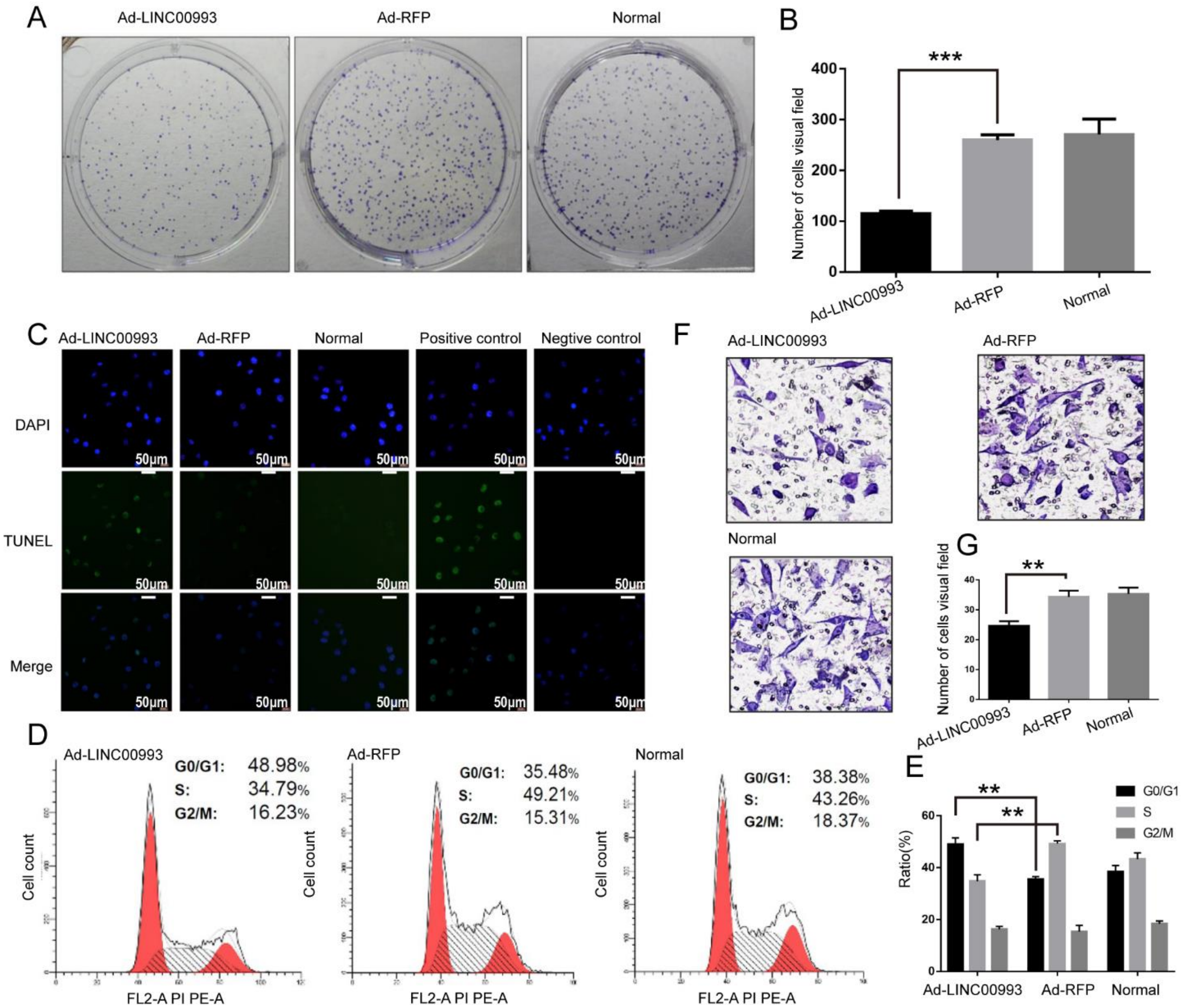

Supplementary Figure S4. LINC00993 suppress the growth of TNBC cells in vitro. (A).Image of clone formation assay. (B). Number of clones were counted 2 weeks after plantation. (C). LINC00993 expression caused apoptosis shown by TUNEL assay. Green points reflected apoptosis, and we used DAPI to stain DNA. Positive control are cells treated with DNase I, negative control are cells collected without adding TUNEL reaction buffer. Original magnification, × 100. Scale bars, 50μm. (D) Effect of LINC00993 on cell cycle detected by flow cytometry. (E) Flow cytometry cell cycle results shown in bar plot. (F).Invasive ability tested by transwell assay. 20000 cells were plated each well, cells were observed after 24 hours of incubation. (G). Bar plot for number of cells migrated across the membrane. ** $P < 0.01$, *** $P < 0.001$, based on Student's t-test. Data were present in M ± SEM.

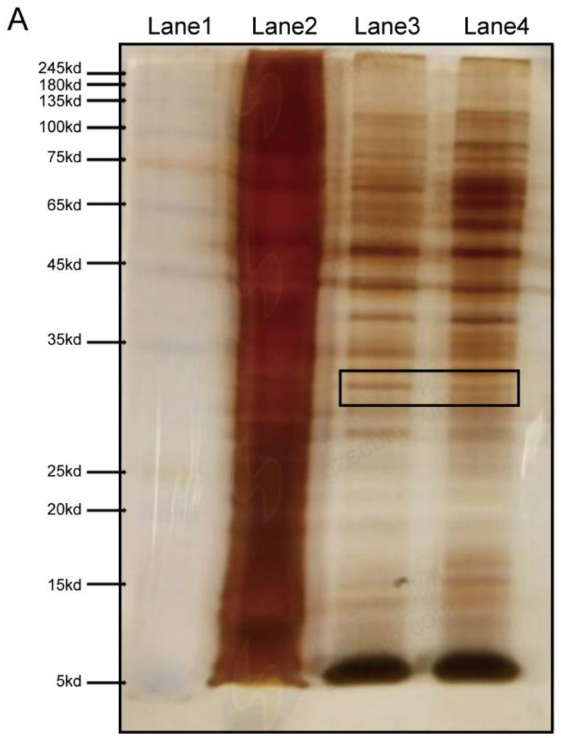

B

Dolton	Polypeptide sequence	Target protein
1698.75	GFAFVTFDDHDSVDK	hnRNPA1
2909.40	IFVGGIKEDTEEHHLRDYFEQYGK	hnRNPA1
1783.90	LFIGGLSFETTDESLR	hnRNPA1
2147.00	GFGFVTYATVEEVDAAMNAR	hnRNPA1
2147.00	GFGFVTYATVEEVDAAMNAR	hnRNPA1

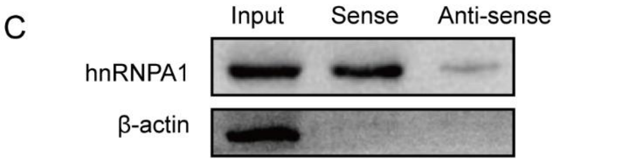

Lane1:marker
Lane2:MDA-MB-231 total protein
Lane3:sense probe +MDA-MB-231 total protein(2mg)
Lane4:anti-sense probe +MDA-MB-231 total protein(2mg)

Supplementary Figure S5. Identification of hnRNPA1 as a Binding Partner for LINC00993. (A). SDS-PAGE analysis of proteins purified from in vitro binding assay using biotinylated LINC00993 or antisense control RNA and MDA-MB-231 protein extracts. The highlighted protein band was subjected to mass spectrometry analysis. (B). Table shows the detected polypeptide sequences from the subjected band by mass spectrometry analysis. (C). Western blot confirms LINC00993 and hnRNPA1 interaction.
